# Bioinformatic meta-analysis reveals novel differentially expressed genes and pathways in sarcoidosis

**DOI:** 10.3389/fmed.2024.1381031

**Published:** 2024-06-13

**Authors:** Rogier T. A. van Wijck, Hari S. Sharma, Sigrid M. A. Swagemakers, Willem A. Dik, Hanna IJspeert, Virgil A. S. H. Dalm, Paul L. A. van Daele, P. Martin van Hagen, Peter J. van der Spek

**Affiliations:** ^1^Department of Pathology & Clinical Bioinformatics, Erasmus MC University Medical Center, Rotterdam, Netherlands; ^2^Laboratory Medical Immunology, Department of Immunology, Erasmus MC University Medical Center, Rotterdam, Netherlands; ^3^Department of Internal Medicine, Division of Allergy & Clinical Immunology, Erasmus MC University Medical Center, Rotterdam, Netherlands

**Keywords:** sarcoidosis, granuloma, gene expression, meta-analysis, IFN-JAK-STAT signalling

## Abstract

**Introduction:**

Sarcoidosis is a multi-system inflammatory disease of unknown origin with heterogeneous clinical manifestations varying from a single organ non-caseating granuloma site to chronic systemic inflammation and fibrosis. Gene expression studies have suggested several genes and pathways implicated in the pathogenesis of sarcoidosis, however, due to differences in study design and variable statistical approaches, results were frequently not reproducible or concordant. Therefore, meta-analysis of sarcoidosis gene-expression datasets is of great importance to robustly establish differentially expressed genes and signalling pathways.

**Methods:**

We performed meta-analysis on 22 published gene-expression studies on sarcoidosis. Datasets were analysed systematically using same statistical cut-offs. Differentially expressed genes were identified by pooling of *p*-values using Edgington’s method and analysed for pathways using Ingenuity Pathway Analysis software.

**Results:**

A consistent and significant signature of novel and well-known genes was identified, those collectively implicated both type I and type II interferon mediated signalling pathways in sarcoidosis. *In silico* functional analysis showed consistent downregulation of eukaryotic initiation factor 2 signalling, whereas cytokines like interferons and transcription factor STAT1 were upregulated. Furthermore, we analysed affected tissues to detect differentially expressed genes likely to be involved in granuloma biology. This revealed that matrix metallopeptidase 12 was exclusively upregulated in affected tissues, suggesting a crucial role in disease pathogenesis.

**Discussion:**

Our analysis provides a concise gene signature in sarcoidosis and expands our knowledge about the pathogenesis. Our results are of importance to improve current diagnostic approaches and monitoring strategies as well as in the development of targeted therapeutics.

## Background

Sarcoidosis is an inflammatory disorder characterized by the formation of non-caseating epithelioid granulomas in various organs. However, the aetiology and pathogenesis of sarcoidosis are not fully understood. The lungs and hilar lymph nodes are most often affected, but almost all organs can be involved (1). The clinical presentation of sarcoidosis is therefore heterogeneous and can range from small benign skin lesions to chronic systemic inflammation. This variability in disease presentation makes it challenging to diagnose sarcoidosis. This diagnosis is mainly based on clinical and radiographic presentation, pathological evidence of non-caseating granulomas and the exclusion of other granulomatous diseases (2, 3). Even though, the lesions resolve spontaneously in a large portion of patients, irreversible tissue damage, like pulmonary fibrosis occurs in up to 20% of cases leading to increased morbidity and mortality (4, 5). More knowledge about the molecular mechanisms involved in the pathophysiology of sarcoidosis is warranted to develop better and more adequate monitoring strategies as well as treatment options.

There is a consensus that complex interactions between genetic and environmental triggers culminate into an aberrant immune response to unidentified antigens including infectious agents (6). Moreover, it is proposed that sarcoidosis encompasses both autoinflammatory and autoimmune features (7). Particularly the similarities of sarcoidosis with Blau syndrome (early-onset sarcoidosis), caused by mutations in nucleotide-binding oligomerization domain containing 2 (*NOD2*), supports an autoinflammatory hypothesis. Meanwhile, the association with *HLA-DRB1* genotypes provides more evidence in the direction of autoimmunity (8). In patients with sarcoidosis, an altered T helper 1 (Th1) immune response is observed partly through activation of signal transducer and activator of transcription 1 (*STAT1*) and production of interferons (IFNs) (9, 10). This has led to the targeting of the Janus kinase (JAK)-STAT signalling pathway in sarcoidosis using inhibitors like baricitinib (11) and tofacitinib (12) for patients with refractory symptoms.

Gene expression studies, also referred to as transcriptomic studies, have been very promising and widely used to identify disease-associated differentially expressed genes (DEGs). Such studies can provide candidate targets for therapy as well as disease biomarkers. However, a concern about transcriptomic studies is their reproducibility and generalizability mainly due to differences in study design, data analysis strategies and limited sample size. With the increasing awareness of open data, more and more datasets are becoming available allowing to identify specific disease associated genes and pathways suitable for therapeutic intervention. Systematic meta-analysis of transcriptomic data for sarcoidosis provides a powerful tool to identify robust gene signatures. Therefore, we systematically analysed sarcoidosis transcriptome by performing meta-analysis on 22 gene expression datasets obtained from various tissues, bronchoalveolar lavage fluid (BALF) and peripheral blood comparing sarcoidosis patients with healthy controls taking into account both the blood and target tissue samples.

## Methods

### Dataset acquisition

The genome expression omnibus (GEO) database (13) was queried for expression profiling by array or high-throughput sequencing using the following string: “Sarcoidosis [All Fields] AND GSE [All Fields].” Datasets containing human RNA expression were selected and further explored with original papers for study design. We excluded single-cell RNA sequencing experiments as well as those with unclear study design or sample annotation. Only datasets with more than four sarcoidosis patients and healthy controls were included in this comparative study. Raw data from these selected studies were downloaded from the GEO database and further processed.

### Dataset preparation and processing

Normalization of Affymetrix and Illumina BeadChip array data was performed with robust microarray average (RMA) within the R package *affy* (14) and with *neqc* within the R package *limma* (15), respectively. Quantile normalization on the gProcessedSignal and subsequent log-transformation, was used for Agilent datasets. RNAseq count data was normalized within the R package *DESeq2* (16). Principal component analysis was performed to assess batch effects and if that was suspected, the *ComBat* function within the *SVA* R package (17) was used for batch correction of the gene expression dataset. Differential expression was calculated for every dataset using the *limma* and *DESeq2* R packages for array and sequencing data, respectively. If a dataset contained multiple cell-types or tissues, it was analysed separately based on the cell-types. A paired analysis was performed on datasets containing multiple samples from a single individual. We did not adjust for confounding factors like age, gender or ethnicity, due to the scarcity of data in the datasets, whereas we aimed at analysing each dataset systematically and uniformly.

### Analysis of datasets for shared genes and signalling pathways

First, we investigated the sarcoidosis datasets for commonly DEGs. In this analysis, we did not differentiate datasets based on cell type or tissue. A list of DEGs was obtained per dataset by setting the significance level of the adjusted *p*-value (*p*_adj_) to less than 0.05 and subsequently annotated the acquired gene lists with HUGO gene symbols. Because the threshold put on the log fold change (FC) depends on the gene and experimental context (18), we did not use the log FC to determine DEGs as a standardized threshold would be too lenient for some datasets and too stringent for others. Ingenuity Pathway Analysis (IPA) (19) was used for functional core analysis of all acquired gene lists. A comparative analysis was performed to investigate the pathways and upstream regulators involved across datasets. Second, the gene lists of DEGs were analysed for overlapping genes. After the individual differential expression analyses, a meta-analysis was performed to test the robustness of our findings. The *p*-values from the individual analyses were combined using Edgington’s method (20) within the R package *metap* (21). The calculated *p*_meta-analysis_ was subsequently Bonferroni corrected to adjust for multiple testing. Pattern of differential expression was investigated for each gene in the individual datasets through the log FC, where a positive and negative log FC were categorized as upregulation and downregulation of the gene, respectively. If the pattern differed in more than three datasets, the gene was not considered consistently differentially expressed and was excluded from further analysis. Thus an acquired gene list was loaded in IPA to build an integrated gene network. Furthermore, to examine these genes in the context of JAK–STAT pathway, IFN signalling was investigated in a separate analysis. These genes were loaded in a dataset (GSE110549) generated by the Immunological Genome Project exploring *in vivo* effects of IFN-α and IFN-γ stimulation on murine macrophages (22).

### Identification of tissue-specific genes and pathways

Furthermore, we investigated whether there are cell type or tissue-specific gene expression profiles in sarcoidosis to determine biological mechanisms of granulomatous inflammation and find specific targets for therapy. Because the majority of datasets contained blood samples, we made a distinction between blood and other cell types. We investigated the acquired gene lists for genes exclusively differentially expressed in the affected sarcoidosis tissues. Finally, identified genes that were exclusively up- or downregulated in tissues were uploaded in IPA to investigate possible tissue-specific pathways, upstream regulators and gene networks.

## Results

### Search and analysis of GEO database

**Figure 1:**
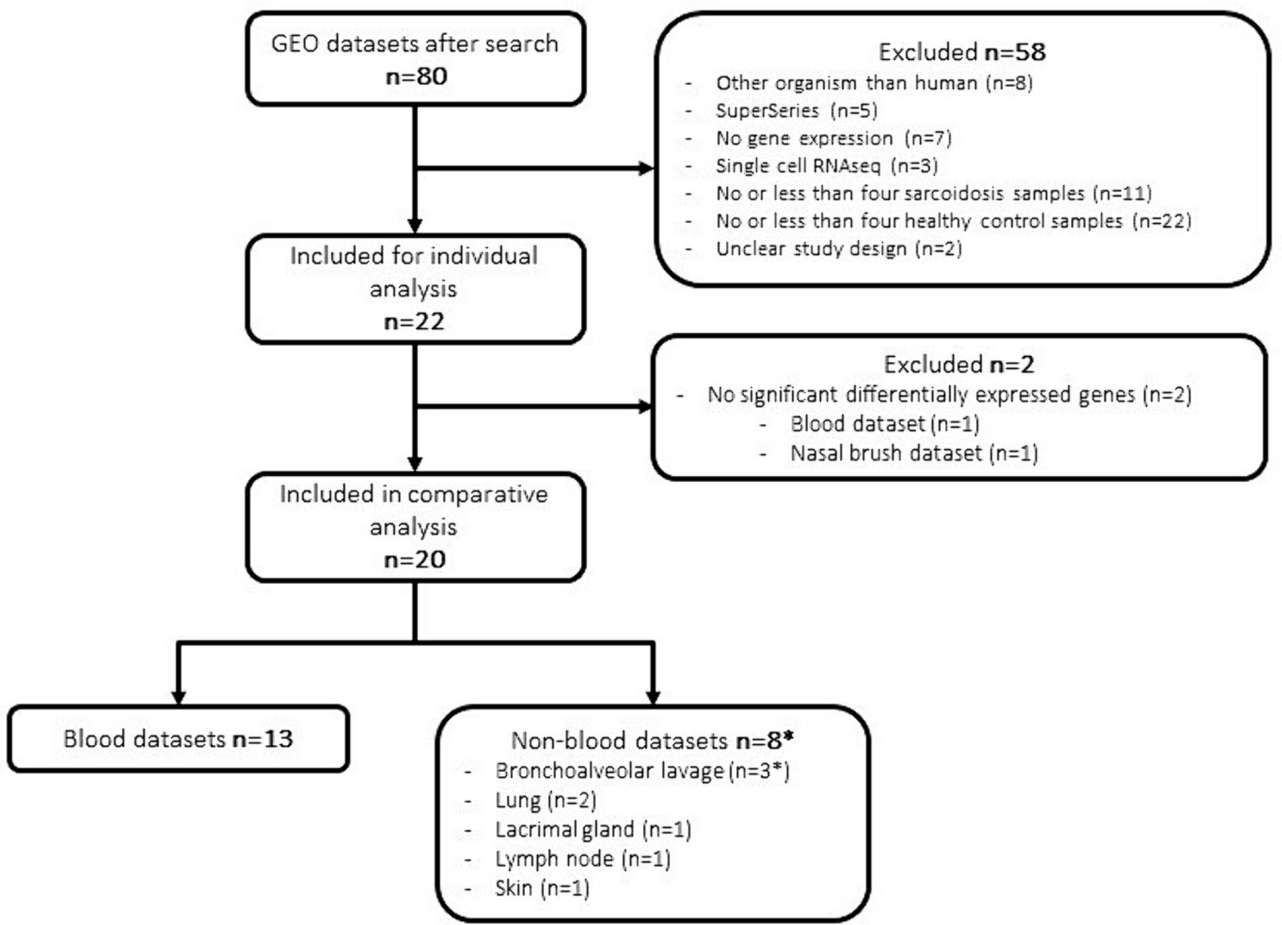
Flowchart of dataset selection and analysis. *One dataset contained both blood and BALF samples.

A systematic search was conducted in the GEO database for sarcoidosis specific datasets. Up until January 2024, our search strategy retrieved 80 datasets worldwide, of which 22 datasets (23–41) were selected and subjected to further analysis after rigorous exclusion criteria (Table 1 and Figure 1). In these 22 datasets, a total of 461 sarcoidosis and 497 healthy control samples across multiple tissues and cell types were analysed. Thirteen datasets used blood derived cells for their analysis, whereas 8 datasets were derived from lung, BALF, nasal brush, lymph node and skin tissue. One dataset contained both blood and BALF samples, which were analysed as separate datasets. Besides diagnostic parameters, clinical details were often not included or reported for individual samples. According to the original manuscripts, 11 out of 22 datasets contained at least one sample that used immunosuppressive therapy for sarcoidosis. Analysis of individual datasets for DEGs (*p*_adj_ < 0.05) revealed altered expression of a large number of genes. Thus derived lists of DEGs per dataset ranged from zero to thousands of genes, including two out of 22 datasets that did not show any DEGs (Figure 1).

**Table 1:** Included datasets and their characteristics.

GSE Accession No	Organism	Technique	Array/Sequencing technology	Tissue	Sarcoidosis (n=)	Control (n=)	Treated samples (at least one)	Reference
GSE83456	Human	Array	Illumina HumanHT-12 V4.0	Whole blood	49	61	Unknown	(24)
GSE37912	Human	Array	Affymetrix Human Exon 1.0 ST	PBMC	39	35	Yes	(37)
GSE19314	Human	Array	Affymetrix Human Genome U133 Plus 2.0	PBMC	38	20	Yes	(31)
GSE42832	Human	Array	lllumina HumanHT-12 V4.0	PBMC subsets	30	30	Yes	(25)
GSE32887	Human	Array	Affymetrix Human Genome U133 Plus 2.0	Skin	26	5	Yes	(30)
GSE73394	Human	Array	Affymetrix Human Gene 1.0 ST	BALF	26	20	Unknown	(34)
GSE42826	Human	Array	Illumina HumanHT-12 V4.0	Whole blood	25	52	Yes	(25)
GSE42830	Human	Array	Illumina HumanHT-12 V4.0	Whole blood	25	38	Yes	(25)
GSE34608	Human	Array	Agilent-014850 Whole Human 4x44K G4112F	PBMC	18	18	Unknown	(33)
GSE75023	Human	Array	Affymetrix Human Genome U133A	BALF	15	12	Yes	(28)
GSE119136	Human	Array	Affymetrix Human Gene 1.0 ST	Nasal brushings	14	12	Yes	(29)
GSE18781	Human	Array	Affymetrix Human Genome U133 Plus 2.0	Whole blood	12	25	Yes	(39)
GSE1907	Human	Array	Affymetrix Human Genome U95A	PBMC	12	12	Unknown	(38)
GSE42825	Human	Array	Illumina HumanHT-12 V4.0	Whole blood	11	23	Yes	(25)
GSE105149	Human	Array	Affymetrix Human Genome U133 Plus 2.0	Lacrimal gland	8	7	Yes	(36)
GSE56998	Human	Array	Affymetrix Human Exon 1.0 ST Array	CD4 T cells	8	8	Unknown	(23)
GSE16538	Human	Array	Affymetrix Human Genome U133 Plus 2.0	Lung	6	6	No	(27)
GSE174659	Human	Sequencing	Illumina HiSeq 4,000	Whole blood & BALF	62	76	Unknown	(32)
GSE155644	Human	Sequencing	Illumina MiSeq	Whole blood	14	14	No	(41)
GSE174443	Human	Sequencing	Ion Torrent Proton	Lymph node	10	7	Unknown	(35)
GSE148036	Human	Sequencing	Illumina HiSeq 3,000	Lung	5	5	No	(26)
GSE192829	Human	Sequencing	Illumina NextSeq 500	PBMC	8	11	No	(40)

### Genes and signalling pathways associated with sarcoidosis

In order to allow cross-platform comparison of the data sets, we annotated the lists of DEGs using the platform identifier with HUGO gene nomenclature. Highly consistent DEGs were identified: 30 genes were differentially expressed in at least 13 datasets, of which 20 showed very consistent pattern of differential expression (if the pattern of differential expression differed in more than three datasets, the gene was not considered as a DEG) across the datasets (Table 2). These genes were differentially expressed in both blood-derived datasets as well as in datasets derived from affected tissues. Guanlytate-binding protein 1 (*GBP1*), *STAT1* and tryptophayl-tRNA synthetase 1 (*WARS1*) showed most consistent differential expression in 16 out of 22 datasets and expression levels were found predominantly upregulated in sarcoidosis. Differential expression of genes high-affinity gamma FC receptor I (*FCGR1A* also referred as CD64), *GBP2*, and vesicle-associated membrane protein 5 (*VAMP5*) was observed in 15 datasets. Interestingly, *STAT1*, *GBP1*, *GBP2* and *GBP5* were upregulated in all datasets (Table 2).

**Table 2:** Differentially expressed genes in at least 13 datasets.

Gene symbol	Frequency differentially expressed	BAL (*n* = 3)	Blood (*n* = 13)	Lung (*n* = 2)	Lacrimal gland (*n* = 1)	Lymph node (*n* = 1)	Nasal brush (*n* = 1)	Skin (*n* = 1)	Number of datasets Upregulated/Downregulated	General directionality in sarcoidosis patients
GBP1	16	2	10	1	1	1	0	0	21/0	Upregulated
STAT1	16	2	10	1	1	1	0	0	21/0	Upregulated
WARS1	16	2	11	0	1	1	0	0	20/1	Upregulated
FCGR1A	15	2	10	0	1	1	0	0	17/3	Upregulated
GBP2	15	1	10	1	1	1	0	0	21/0	Upregulated
VAMP5	15	2	10	1	1	0	0	0	20/1	Upregulated
GBP4	14	1	9	1	1	1	0	0	18/1	Upregulated
GBP5	14	1	10	0	1	1	0	0	19/0	Upregulated
LAP3	14	1	9	1	1	1	0	0	18/2	Upregulated
PSTPIP2	14	2	9	0	1	1	0	0	19/1	Upregulated
TAP1	14	2	9	1	1	0	0	0	19/2	Upregulated
ABLIM1	13	2	9	0	1	0	0	0	3/18	Downregulated
ANKRD22	13	1	9	0	1	1	0	0	18/1	Upregulated
C2	13	1	8	1	1	1	0	0	18/3	Upregulated
GADD45B	13	1	10	0	1	0	0	0	18/3	Upregulated
HNRNPDL	13	1	10	1	0	1	0	0	3/18	Downregulated
IRF1	13	2	8	0	1	1	0	0	19/2	Upregulated
MAFB	13	2	8	0	1	1	0	0	19/1	Upregulated
STX11	13	1	9	0	1	1	0	0	20/1	Upregulated
TYMP	13	2	9	0	1	1	0	0	19/2	Upregulated

List of genes that were differentially expressed in at least twelve datasets comparing sarcoidosis samples to healthy controls and which showed consistent directionality of differential expression across the datasets. Information by tissue type is incorporated in this table.

**Figure 2:**
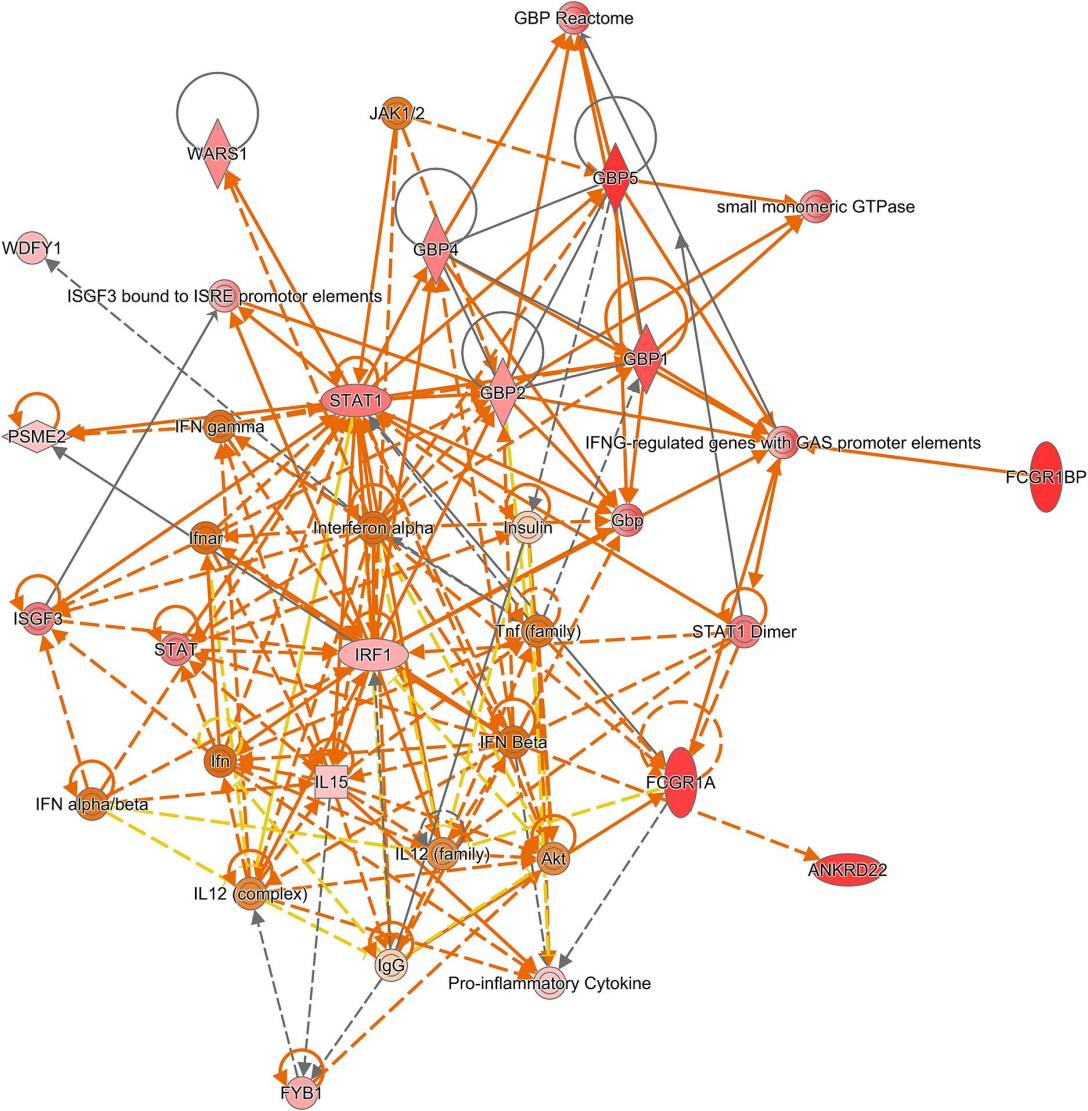
Connectivity network generated on 16 of the 25 significant genes from the meta-analysis. Centrally positioned in the network are *STAT1* and interferon alpha, which suggests these play an important role in sarcoidosis. Red and green molecules are up- and downregulated in the genelist respectively, whilst orange and blue are predicted to be up- and downregulated based on the input genelist by the molecule activity predictor (MAP) function within IPA.

Next, we integrated all the datasets to perform meta-analysis on the *p*-values to identify additional consistent DEGs as some genes could not be investigated on all transcriptomic platforms due to their varied design. Pooling of *p*-values with Edgington’s method and subsequent Bonferroni correction resulted in 36 significantly expressed genes. Of these 36 genes, 12 displayed variable pattern of up or down regulation and hence, they were excluded from further analysis (Table 3). To investigate the relationship between the remaining 24 genes, a connectivity plot was generated in IPA (Figure 2). Remarkably, more than half (14 out of the 24) of the genes were well connected in the connectivity network. *STAT1*, IL-12, and IFN signalling pathways appeared centrally positioned within the network and were predicted to be activated. Also insulin was positioned in the network. Both type I IFN (IFN-α and IFN-β) and type II IFN (IFN-γ) were predicted to be upregulated within the connectivity network. Therefore, we explored this further in a murine dataset specifically on IFN signalling (GSE110549) where we verified that half of the genes identified in the meta-analysis were upregulated upon IFN stimulation, advocating for a pivotal role of altered IFN signalling in sarcoidosis (Supplementary Figure S1).

**Table 3:** Gene list of the significant (*p*_adj_ < 0.05) genes in the meta-analysis.

Gene symbol	Adjusted *p*-value	Number of datasets Upregulated/Downregulated
STAT1	9.27E-07	21/0
WARS1	1.26E-06	20/1
GBP1	1.79E-05	21/0
VAMP5	5.35E-05	20/1
PSTPIP2	7.20E-05	19/1
ANKRD22	9.75E-05	18/1
GBP5	0.000169	19/0
FCGR1A	0.000452	17/3
GBP2	0.000827	21/0
FCGR1B	0.001204	13/2
STX11	0.001292	20/1
PSME2	0.001619	20/1
UBE2L6	0.002172	20/1
GBP4	0.002705	18/1
PLEK	0.003109	19/2
CALHM6	0.005246	17/0
DHRS9	0.005825	18/2
IL15	0.014057	19/2
SOD2	0.0171	20/0
IRF1	0.0262	19/2
GLUL	0.027109	18/3
LAP3	0.03706	18/2
WDFY1	0.043271	19/0
FYB1	0.049856	19/2

Adjusted *p*-value is Bonferroni corrected. The direction of the differentially expressed gene is determined by investigating the directionality of fold change in the individual datasets. A gene was not considered significant if the directionality differed in more than three datasets from the main directionality.

### *In silico* functional analysis for the signalling pathways

**Figure 3:**
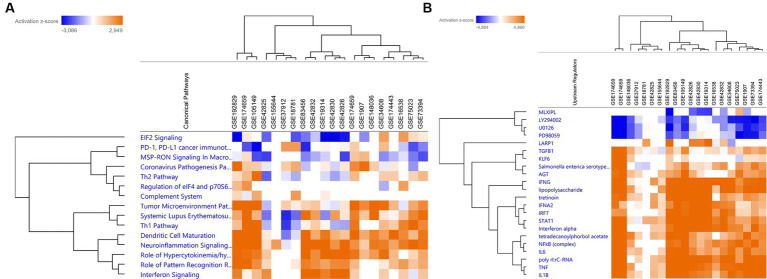
*In silico* functional analysis of the individual differentially expressed gene lists. **(A)** Heatmap of the comparison analysis within IPA that shows the canonical pathways involved in each individual dataset. Across the datasets, similar canonical pathways were predicted to be involved. **(B)** Heatmap that shows the upstream regulators predicted to be involved across the functional analyses of the individual datasets.

All individual gene lists of DEGs were loaded into IPA for functional pathway analyses. Eukaryoitc initation factor 2 (EIF2) signalling pathway was consistently predicted to be downregulated across datasets in sarcoidosis patients as compared to healthy controls. On the other hand, pro-inflammatory pathways like IFN-signalling, neuro-inflammation and hypercytokinaemia/hyperchemokinaemia were predicted to be activated in sarcoidosis patients (Figure 3A). This was further indicated by the upstream regulators found within IPA analysis. Pro-inflammatory transcription factors and cytokines (i.e., *STAT1*, TNF, IL-6 and IL-1β) were consistently predicted to be activated (Figure 3B). Also type I and type II IFNs were consistently predicted to be upregulated, strongly suggesting activation of the IFN-STAT1 pathway. Only a few upstream regulators were consistently downregulated after stringent filtering within IPA. Inhibitors like, PD98059 U0126 and LY294002 (Figure 3B) target the MAPK/ERK and PI3K/AKT/mTOR pathways, suggesting that these chemical compounds could inhibit these activated signalling pathways in sarcoidosis. These pathways often run in parallel and converge to regulate important cellular processes.

### Identification of tissue-specific DEGs

**Figure 4:**
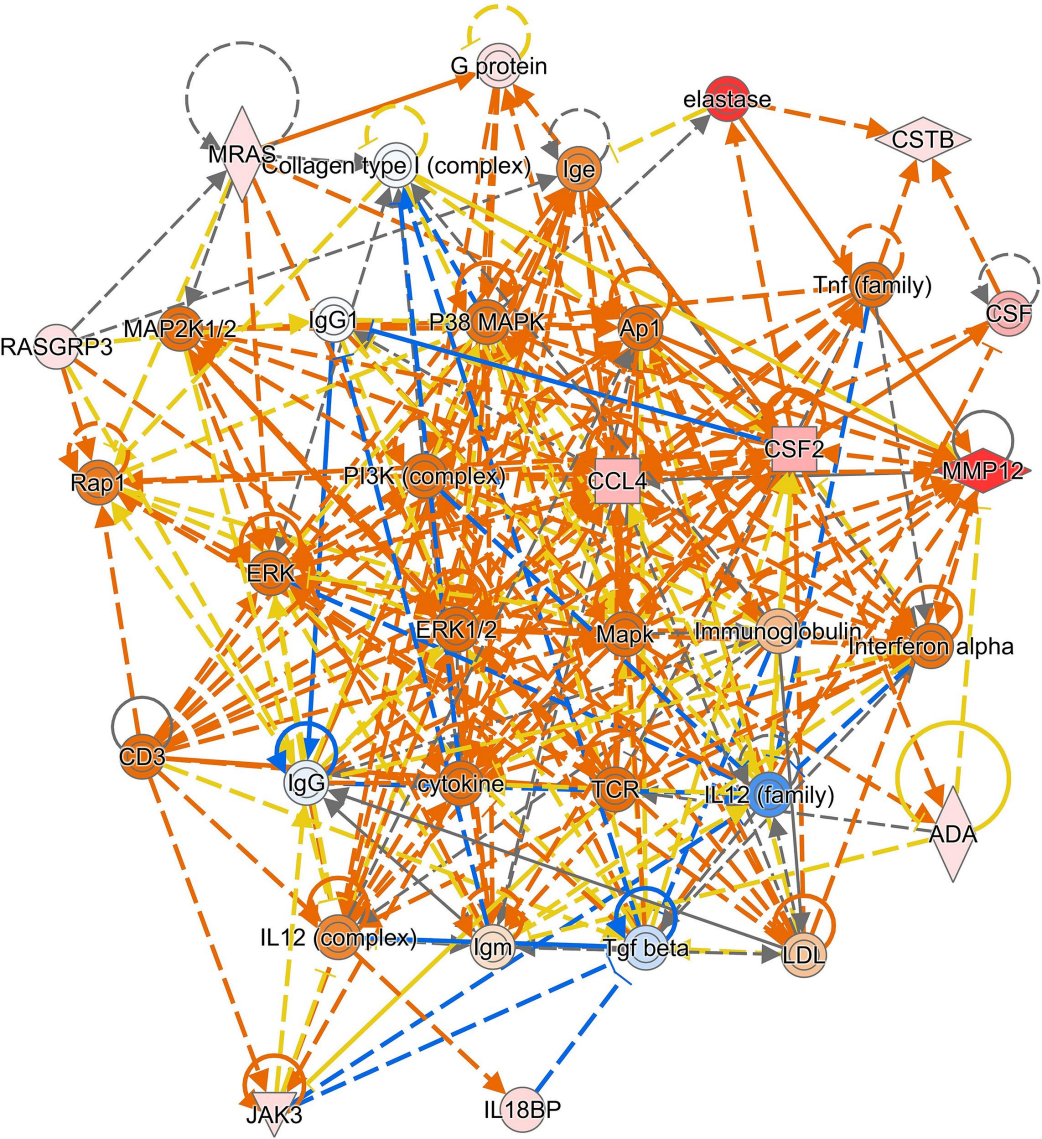
Connectivity network generated on 9 of the 14 genes with high tissue specificity. The PI3K-pathway and MAPK/ERK signalling are centrally positioned in the network. Red and green molecules are up- and downregulated in the genelist respectively, whilst orange and blue are predicted to be up- and downregulated based on the input genelist by the molecule activity predictor (MAP) function within IPA.

Finally, we investigated tissue-specific gene signatures in sarcoidosis. We hypothesized that DEGs in affected tissues may play a crucial role in granuloma formation and their maintenance. Nine datasets were retrieved from tissues, of which 8 showed DEGs. A disintegrin and metalloprotease like decysin 1 (*ADAMDEC1*) and WD repeat and SOCS box-containing protein 1 (*WSB1*) were differentially expressed (upregulated) in seven datasets, however, these were also differentially expressed in a few blood datasets. The most consistent tissue-specific DEG was matrix metallopeptidase 12 (*MMP12*), which was highly upregulated in 6 tissue-derived datasets (two BALF, two lung, one lacrimal gland and one lymph node derived dataset) and in no blood-derived dataset. In five of the tissue-specific datasets C-X-C motif chemokine receptor 6 (*CXCR6*) and syntrophin beta 2 (*SNTB2*), whereas in four datasets colony stimulating factor 2 (*CSF2*), fatty acid desaturase 1 (*FADS1*), interleukin 18 binding protein (*IL18BP*), acyl-CoA synthetase family member 2 (*ACSF2*), cystatin B (*CSTB*), C-C motif chemokine ligand 4 (*CCL4*), adenosine deaminase (*ADA*), *JAK3*, malic enzyme 1 (*ME1*), muscle RAS Oncogene Homolog (*MRAS*) and RAS guanyl releasing protein 3 (*RASGRP3*) were differentially expressed. Most of these genes are involved in the activation of processes such as leukocyte recruitment and migration and form a concise network around PI3K and MAPK/ERK signalling (Figure 4).

## Discussion

Using public datasets in the GEO database, we studied 22 sarcoidosis datasets and performed a meta-analysis to identify genes and pathways those are common across these studies and differentiate sarcoidosis patients from healthy controls. We found that there are clear differences in gene expression profiles for *GBP1*, *STAT1* and *WARS1* among others between sarcoidosis patients and healthy controls. Furthermore, the integration of datasets provided a comprehensive view for certain genes (*STAT1, WARS1, GBP1, VAMP5*, and *PSTPIP2*) being consistently expressed in the majority of datasets advocating for their role in the pathogenesis of sarcoidosis. These genes could potentially be used to develop meaningful genomic-derived biomarkers for sarcoidosis. In analogy to several other transcriptomic studies, we identified both type I and type II IFN signalling as important pathways (42), but in this meta-analysis also poorly studied pathways became apparent in the context of sarcoidosis such as EIF2 signalling and neuro-inflammation. Finally, we explored datasets with samples from sarcoidosis-affected tissues and identified tissue-specific DEGs (*MMP12*, *CXCR6*, and *SNTB2*) those likely to play important respective roles, specifically in granulomatous inflammation. Hence, it can be proposed that these specific genes and their respective translated proteins could be targeted for precise therapy of sarcoidosis lesions.

Whether a gene is considered differentially expressed in transcriptomic studies revolves around the chosen cut-offs in (adjusted) *p*-value and fold change. The results between transcriptomic studies often differ due to differences in study design, analysis strategies and sample size. Therefore, to circumvent these issues, a meta-analysis on several datasets of particular disease, becomes an important tool for analysis and inferences increasing the strength of such studies to establish true signals (43). We assessed the differential expression pattern by integrating these datasets based on the *p*-values thus creating a robust analysis to identify consistently DEGs. In this study, to the best of our knowledge, the largest systematic meta-analysis of transcriptomic data in sarcoidosis is being presented.

Sarcoidosis research so far has predominantly focused on the IFN-STAT1 pathway linked to T cells and macrophages. In fact, there have been numerous reports on the development and exacerbation of sarcoidosis after IFN therapy (44, 45). Our study highlights the importance of this pathway as several of the most consistently DEGs are associated with IFN signalling. Furthermore, in IPA analysis JAK1/2 was found in the connectivity network, strongly suggesting the rationale to target the JAK-STAT pathway with JAK inhibitors in sarcoidosis patients. Interestingly, we identified multiple members of GBP gene family to be consistently differentially expressed (*GBP1, GBP2, GBP4 and GBP5*). These genes are a group of seven IFN-inducible GTPases implicated in the host defence against intracellular pathogens by targeting and inducing lysis of pathogen-containing vacuoles (46). Differential expression of GBPs as found in our study, is not only attributed to the pulmonary sarcoidosis, but aberrant expression of *GBP1* has also been demonstrated earlier in acute respiratory distress syndrome (47).

One of the most consistently differentially expressed genes was *FCGR1A* (CD64), which was differentially expressed in 15 out of 22 datasets. This gene is strongly induced by IFN-γ and plays a central role in antibody-dependent cytotoxicity and FCγ receptor-mediated phagocytosis (48). Phagosome and phagocytosis has been reported to be upregulated in monocytes of sarcoidosis patients (49). In proteomic studies, FCγ receptor-mediated phagocytosis is upregulated in sarcoidosis (50, 51). Several genes including *FCGR1A*, ubiquitin conjugating enzyme E2 L6 (*UBE2L6*) and *VAMP5*, found statistically significant in our meta-analysis have also been described in the context of tuberculosis (TB) (52). In both sarcoidosis and TB, granulomas are the hallmark, but the granulomas in sarcoidosis are non-caseating, whereas the granulomas in TB frequently contain a necrotic core. Most likely these diseases, despite their differences, may share common inflammatory pathways and mechanisms corroborated by overlapping gene expression profiles (25, 31, 33).

Our analysis revealed novel genes, such as *WARS1* and *VAMP5* those were never implicated in sarcoidosis. *WARS1* is an essential enzyme called tryptophayl-tRNA synthetase 1 that charges tryptophane to its cognate tRNA and also plays a role in the innate immune system. *WARS1* is upregulated upon infection and can act as a ligand of toll-like receptor (TLR) 2 and TLR4. This leads to secretion of cytokines and activation of various immune pathways (53). *VAMP5* is part of the SNARE protein family, which is involved in vesicle fusion and recycling (54). *VAMP5* is involved in intracellular transport including exocytosis, endocytosis and recycling of endosomes (55). These processes are closely related to autophagic pathways, which have been implicated in the pathogenesis of sarcoidosis (56). Identification of these genes adds to the knowledge about the genetics and pathogenesis of sarcoidosis and opens avenues for further research into these proteins.

IPA analysis of datasets analysed in this study predicted EIF2 signalling to be downregulated. The EIF2 signalling cascade is involved in autophagy, protein translation and cell survival as well as the mammalian target of rapamycin (mTOR) pathway (57, 58). Recently, the mTOR pathway gained much attention in sarcoidosis (59–61), after the finding that constitutive activity of mTORC1 causes formation of granulomas (62). In this regard, Gupta and colleagues successfully treated a patient with pulmonary sarcoidosis with mTOR inhibitor sirolimus (63). Moreover, our tissue-specific analysis showed PI3K in the connectivity network, suggesting that the PI3K/mTOR pathway is involved only in affected tissues. Another interesting finding in IPA analysis was the upregulation of neuro-inflammation signalling pathway. Small fiber neuropathy is observed in about 30% of patients with systemic sarcoidosis (64), in which circulating inflammatory and neurotoxic cytokines may be involved (65). Recently, the upregulation of the neuro-inflammatory response was found by another group studying the sarcoidosis transcriptome and proteome (66). This interesting study is a meta-analysis as well, however, their strategy differed significantly from ours as they performed meta-analysis on the common pathways rather than DEGs. Moreover, recently two other meta-analyses on the transcriptome of sarcoidosis have been published (67, 68). These studies used only 11 and 13 studies respectively, excluding many relevant studies in their analyses. Therefore, our study is the largest systematic meta-analysis to identify robust DEGs which can be of value as biomarkers for sarcoidosis.

Interestingly, insulin was another hub in the connectivity network generated by IPA. Sarcoidosis patients are at increased risk for developing type 2 diabetes (T2D) (69). Much is unknown about this association, however, chronic inflammation and increased secretion of cytokines might predispose sarcoidosis patients to develop T2D (70). The IFN-γ/STAT1 upregulation, as found in our study, could be linked to insulin resistance in adipocytes through multiple mechanisms, including downregulation of the insulin receptor and glucose transporter type 4 (71). Additionally, *STAT1* expression in white adipose tissue is elevated in prediabetic patients and STAT1 levels are positively correlated with plasma glucose (72). Together, these data suggest an important role for IFN-γ/STAT1 signalling in T2D observed in sarcoidosis patients, that is further exacerbated by steroid treatment and warrants attention from clinicians.

Finally, we looked for a tissue-specific gene signature in sarcoidosis and found *MMP12* upregulated in most tissue-specific datasets. *MMP12* is an elastase enzyme predominately produced by M2 tissue macrophages (73), those aggregate and form the characteristic multinucleated giant cells as seen in granulomas (74). Involvement of *MMP12* in sarcoidosis specially in granuloma progression has been reported previously (27, 75). In our study, *MMP12* was only differentially expressed at tissue sites, which is highly suggestive for its crucial role in granuloma formation. *CXCR6* was also tissue-specific in our analysis, and this gene has been found to be expressed in Th1 cells surrounding the central core of sarcoidosis granulomas (76). Therefore, tissue-specific DEGs like *MMP12* and *CXCR6*, or potentially its ligand *CXCL16*, could be interesting therapeutic targets for sarcoidosis lesions.

### Limitations of the study

Among 22 datasets included in this meta-analysis, we were limited with respect to sample size, variable tissues and cell types as well as different technological platforms. Additionally, sarcoidosis patients can differ substantially in clinical presentation, disease progression and treatment response. Studies have shown distinct gene expression profiles between self-limiting sarcoidosis and progressive sarcoidosis (37, 77). Unfortunately, clinical information such as age, sex, ethnicity, treatment regimen, and disease activity were poorly reported by most studies. To appropriately circumvent these limitations, we opted for an iterative and systematic approach, through which we observed highly consistent DEGs and pathways in sarcoidosis despite this variability among studies. Furthermore, we were not able to adjust for other confounding factors such as lymphopenia, which is often observed in sarcoidosis patients and associated with disease activity (78). Attention should be paid to treatment regime and disease activity. Eleven of the 22 included datasets contained at least one sample that used immunosuppressive therapy, whereas three datasets excluded patients who were on immunosuppression. We did not observe major differences in DEGs between these datasets, but it is known that immunosuppression can alter gene expression in disease (79). Appropriate studies with disease endotyping are needed to study and identify potential biomarkers stratifying sarcoidosis subgroups leading to a precision-medicine approach (80). Finally, only eight of the 22 studies investigated here were derived from target tissues, highlighting the need for more studies investigating tissue-specific signatures to gain more insight in the genes and pathways involved in granuloma formation. Whether the described genes in this study represent activation or perpetuation of the disease needs further exploration.

## Conclusion

In this meta-analysis study, 22 sarcoidosis gene expression datasets were systematically and uniformly assessed to identify DEGs and their signalling pathways. Integration of the results from individual datasets revealed a number of novel candidate genes (i.e., *GBPs*, *VAMP5* and *WARS1*) and pathways in addition to previously described DEGs in sarcoidosis. Meta-analysis identified a robust and compact gene signature that points towards altered IFN-JAK-STAT1 signalling in sarcoidosis. Our findings add to the emerging evidence to employ JAK inhibitors as a targeted treatment in sarcoidosis patients. More strikingly, the DEGs found in our meta-analysis can further be explored to develop genomic-derived biomarkers for sarcoidosis. We found tissue-specific signature of genes like *MMP12*, *CXCR6*, and *SNTB2* suggesting their pathways are likely to be involved in granuloma formation and progression and could eventually be potential therapeutic targets for sarcoidosis. Clinical manifestation still remains a challenge with respect to disease activity and progression, which warrants the need for further transcriptomic studies with endotyping investigating pulmonary phenotypes and immune responses.

## Data availability statement

The original contributions presented in the study are included in the article/Supplementary material, further inquiries can be directed to the corresponding author.

## Ethics statement

Ethical approval was not required for the studies involving humans because the current study uses existing publicly available data from gene expression studies, which obtained consent and ethical approval. No new data was generated in this study. Ethical approval was therefore not required. The studies were conducted in accordance with the local legislation and institutional requirements. The human samples used in this study were acquired from published publicly available datasets. Written informed consent to participate in this study was not required from the participants or the participants’ legal guardians/next of kin in accordance with the national legislation and the institutional requirements.

## Author contributions

RW: Writing – review & editing, Writing – original draft, Methodology, Investigation, Formal analysis, Data curation, Conceptualization. HS: Writing – review & editing, Writing – original draft, Investigation. SS: Writing – review & editing, Writing – original draft, Investigation, Formal analysis, Data curation. WD: Writing – review & editing, Writing – original draft, Investigation. HI: Writing – review & editing, Writing – original draft, Investigation. VD: Writing – review & editing, Writing – original draft, Investigation. PD: Writing – review & editing, Writing – original draft, Investigation. PH: Writing – review & editing, Writing – original draft, Investigation, Conceptualization. PS: Writing – review & editing, Writing – original draft, Investigation, Formal analysis, Data curation, Conceptualization.

## Figures and Tables

**Table:** 

ADAMDEC1	a disintegrin and metalloprotease like decysin 1
ACSF2	Acyl-CoA synthetase family member 2
ADA	adenosine deaminase
BALF	bronchoalveolar lavage fluid
CCL4	C-C motif chemokine ligand 4
CSF2	colony stimulating factor 2
CSTB	Cystatin B
CXCR6	C-X-C motif chemokine receptor 6
DEG	differentially expressed gene
EIF2	Eukaryoitc initation factor 2
FADS1	fatty acid desaturase 1
FC	fold change
FCGR1A	high-affinity gamma FC receptor I
GEO	genome expression omnibus
GBP	guanylate-binding protein
IFN	interferon
IL18BP	interleukin 18 binding protein
IPA	ingenuity pathway analysis
JAK	Janus kinase
ME1	malic enzyme 1
mTOR	mammalian target of rapamycin
MMP12	matrix metallopeptidase 12
MRAS	muscle RAS oncogene homolog
NOD2	nucleotide-binding oligomerization domain containing 2
RASGRP3	RAS guanyl releasing protein 3
RMA	robust microarray average
STAT1	signal transducer and activator of transcription 1
SNTB2	syntrophin beta 2
WARS1	Tryptophayl-tRNA synthetase 1
T2D	type 2 diabetes
TB	tuberculosis
Th1	T helper 1
TLR	toll-like receptor
UBE2L6	ubiquitin conjugating enzyme E2 L6
VAMP5	vesicle-associated membrane protein 5
WSB1	WD repeat and SOCS box-containing protein 1
