# Supplementary figures and images for: Bioinformatic meta-analysis reveals novel differentially expressed genes and pathways in sarcoidosis
Source: Front Med (Lausanne). 2024 Jun 13;11:1381031. doi: 10.3389/fmed.2024.1381031 (PMC11208482; doi:10.3389/fmed.2024.1381031)

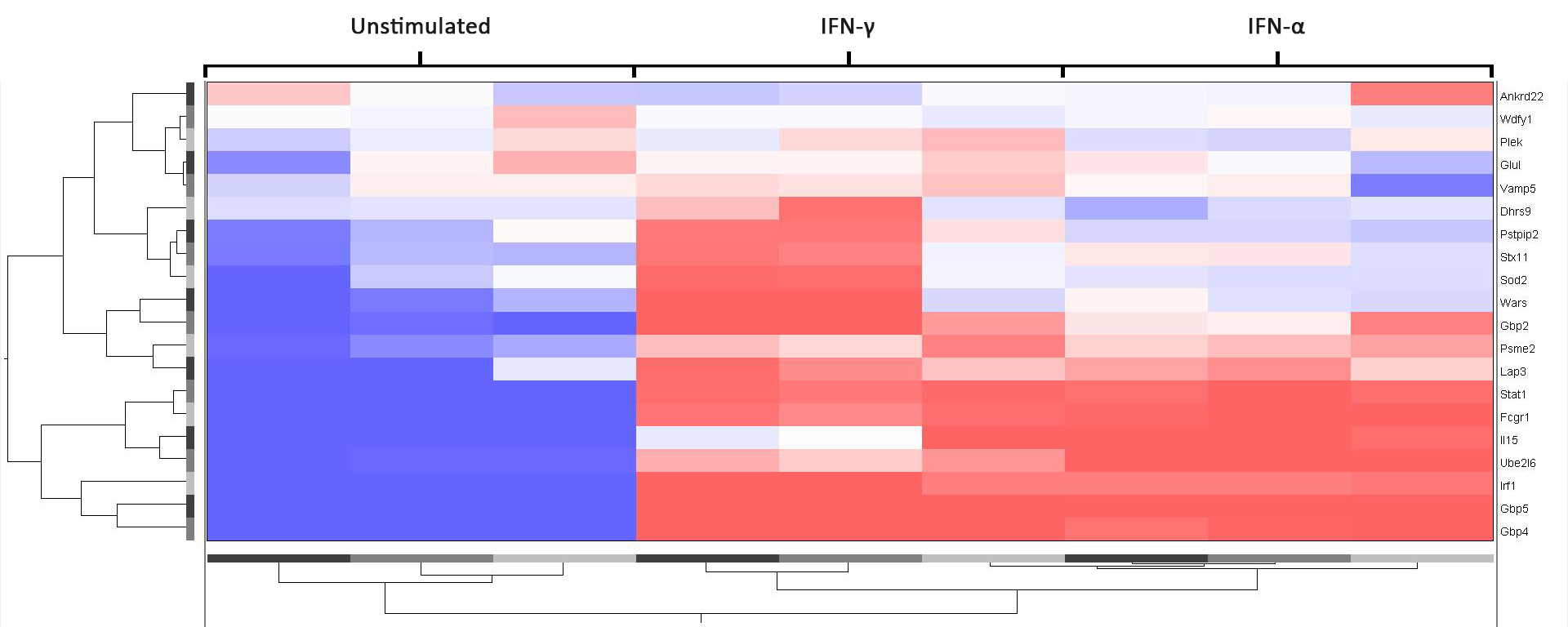

Supplement: Supplementary file 1 [file Image_1.JPEG]
